# Supplementary material for: Efficacy of a respiratory syncytial virus vaccine candidate in a maternal immunization model
Source: Nat Commun. 2018 May 15;9:1904. doi: 10.1038/s41467-018-04216-6 (PMC5953919; doi:10.1038/s41467-018-04216-6)
Supplement: Supplementary file 1 — Supplementary Information [file 41467_2018_4216_MOESM1_ESM.pdf]

Efficacy of a respiratory syncytial virus vaccine candidate in a maternal immunization model

Blanco et al.

Supplementary Figures

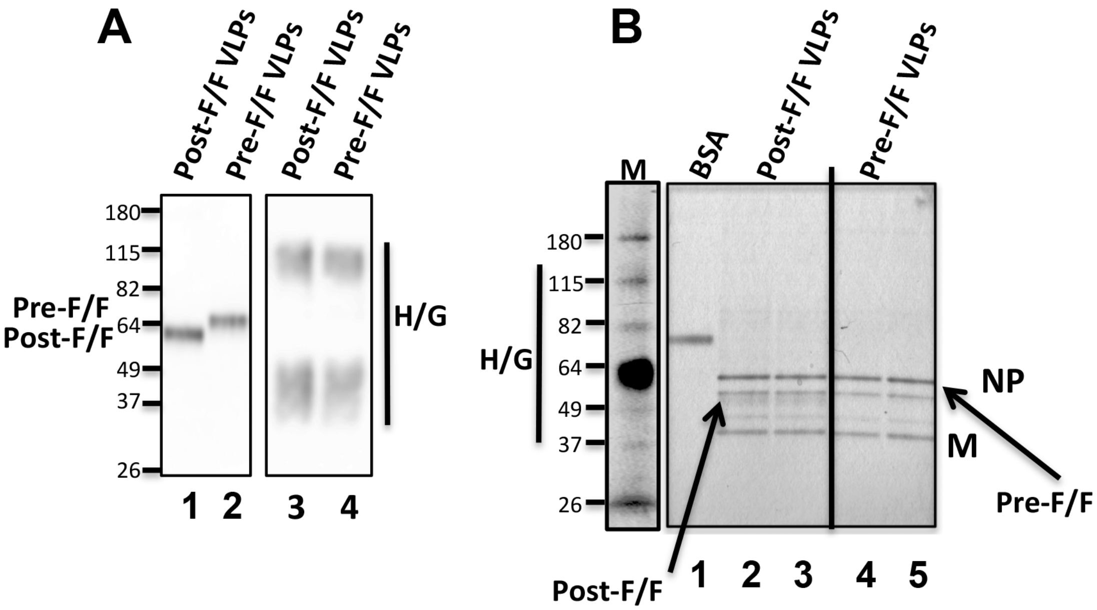

Supplementary Figure 1: *Proteins in the VLPs*

(A) Shown are Western blots of polyacrylamide gels containing equivalent amounts of total protein of VLP-H/G+Post-F/F (lanes 1 and 3) and VLP-H/G+Pre-F/F (lanes 2 and 4) (100 ng total protein/lane). Blots in lanes 1 and 2 were incubated with anti-RSV F protein HR2 antibody while blots in lanes 3 and 4 were incubated with anti-G peptide antibody. (B) Shown is a silver stain of proteins in duplicate lanes in polyacrylamide gels of equivalent amounts of VLP-H/G+Post-F/F (lanes 2 and 3) and VLP-H/G+Pre-F/F (lanes 4 and 5) (100 ng total protein/lane). NP, NDV nucleocapsid protein; M, NDV membrane protein. Marker BSA is shown in lane 1. Polyacrylamide gels for Western blots and silver staining were 8% Bis-Tris polyacylamide gels run with MOPS Running Buffer. The molecular weight standards, run in parallel on each gel, were Benchmark protein standards

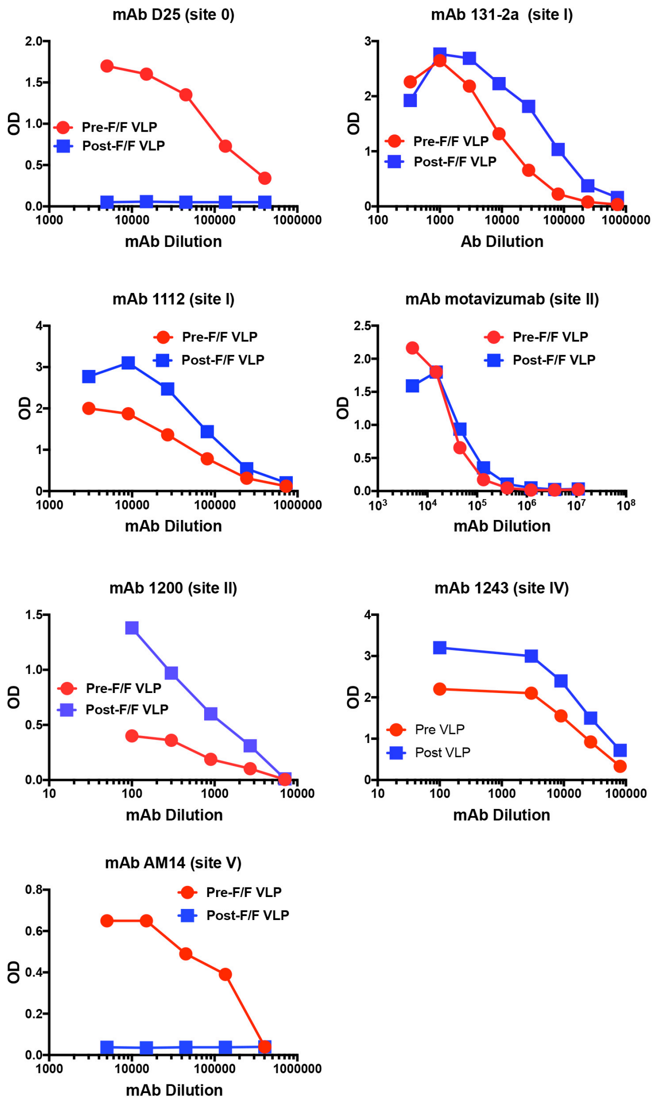

Supplementary Figure 2: *Protein Monoclonal Antibody Binding to VLPs*

Equivalent amounts of VLP-H/G+Post-F/F (blue) and VLP-H/G+PreF/F (red) were bound to microtiter wells (37.5 ng F/well) as previously described[7]. Wells were incubated with increasing amounts of seven different anti-F protein monoclonal antibodies specific for representative sites on the F protein[26, 29-32] and binding was detected as previously described[7]. mAb D25 and mAb AM14 are specific for sites present only on the pre-fusion F protein[26, 31, 32].

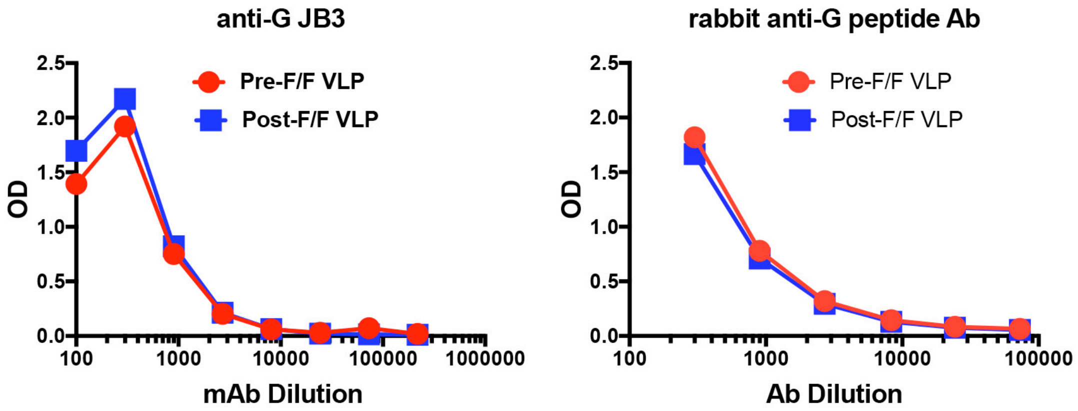

Supplementary Figure 3: *G protein Antibody Binding to VLPs* Equivalent amounts of VLP-H/G+Post-F/F (blue) and VLP-H/G+PreF/F (red) were bound to microtiter wells as previously described (100 ng total protein/well). Wells were incubated with increasing amounts of mAb 1189 (obtained from J. Beeler) or rabbit anti-G peptide antibody (ThermoFisher PA5-22827) and binding of antibodies was detected as previously described[7].

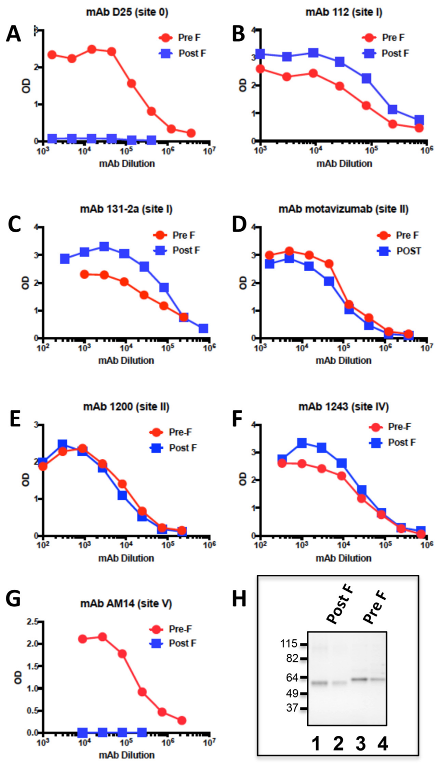

Supplementary Figure 4: *Characterization of Purified Soluble F Proteins***.** (A-G) Equivalent amounts of soluble post-F (blue) and soluble pre-F (red) proteins were bound to Ni coated microtiter wells (37.5 ng F/well), in order to bind proteins by their his-tags, as previously described[7]. Wells were incubated with increasing amounts of seven different anti-F protein monoclonal antibodies specific for representative sites on the F protein[26, 29-32] and binding was detected as previously described[7]. (H) Silver stain of purified soluble post-F (lanes 1, 2) and purified soluble pre-F (lanes 3, 4). Lanes 1 and 3, 25 micrograms F protein; Lanes 2 and 4, 12.5 micrograms of protein. Polyacrylamide gels for Western blots and silver staining were 8% Bis-Tris polyacylamide gels run with MOPS Running Buffer. The molecular weight standards, run in parallel on each gel, were Benchmark protein standards.

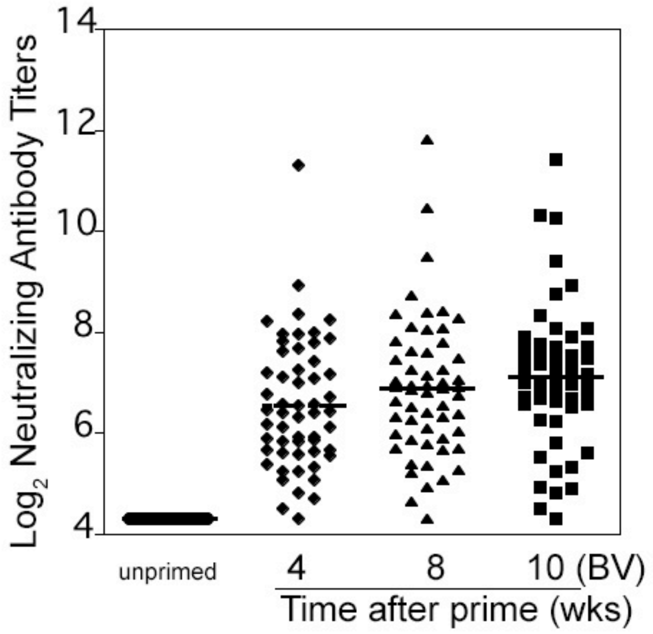

Supplementary Figure 5: *Serum NA of the female cotton rats at 4, 8, or 10 weeks (before vaccination, BV) post-priming.* Each symbol represents a female cotton rat (n=55) and the cross bar represent mean neutralizing antibodies.

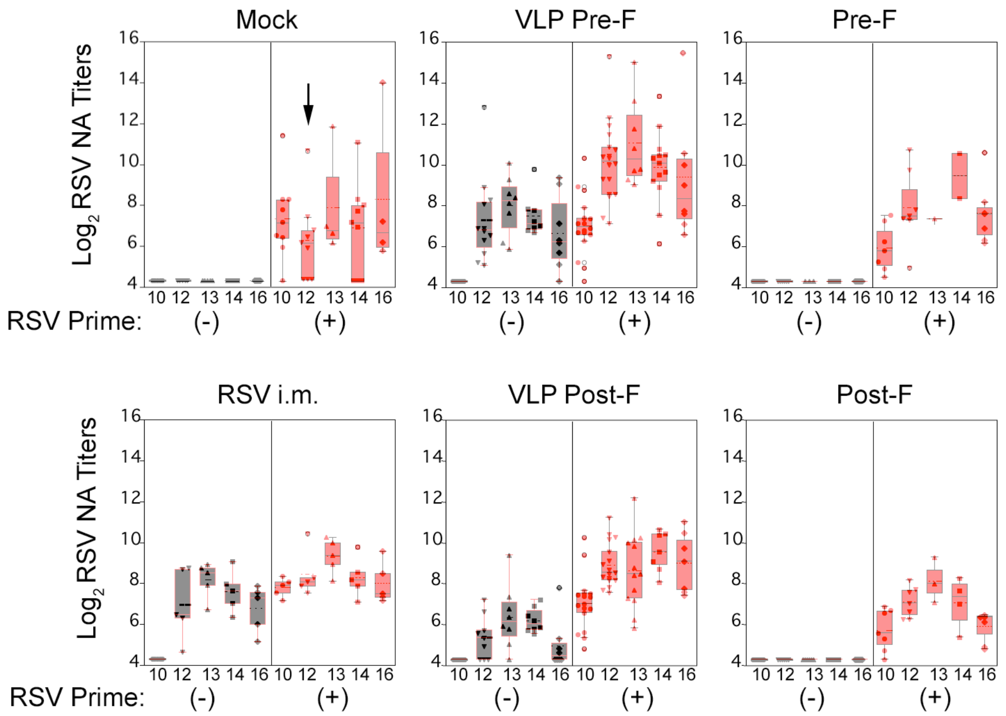

Supplementary Figure 6: *RSV serum neutralizing antibodies measured in females at the indicated days post-priming.* RSV primed (red symbols) or unprimed (black symbols) females were bled at the indicated times post-priming. Week 10 samples represent sera taken before vaccination whereas week 12 samples represent sera taken before delivery of pups. Symbols represent individual dams. Bar across represent the mean ± s.e.m of a group. Boxes represent the boundaries of the quartiles. Each graph corresponds to a single vaccination group. Horizontal black lines represent mean value. Arrow indicates the drop in NA before delivery, seen only in primed, unvaccinated animals and previously described[9].

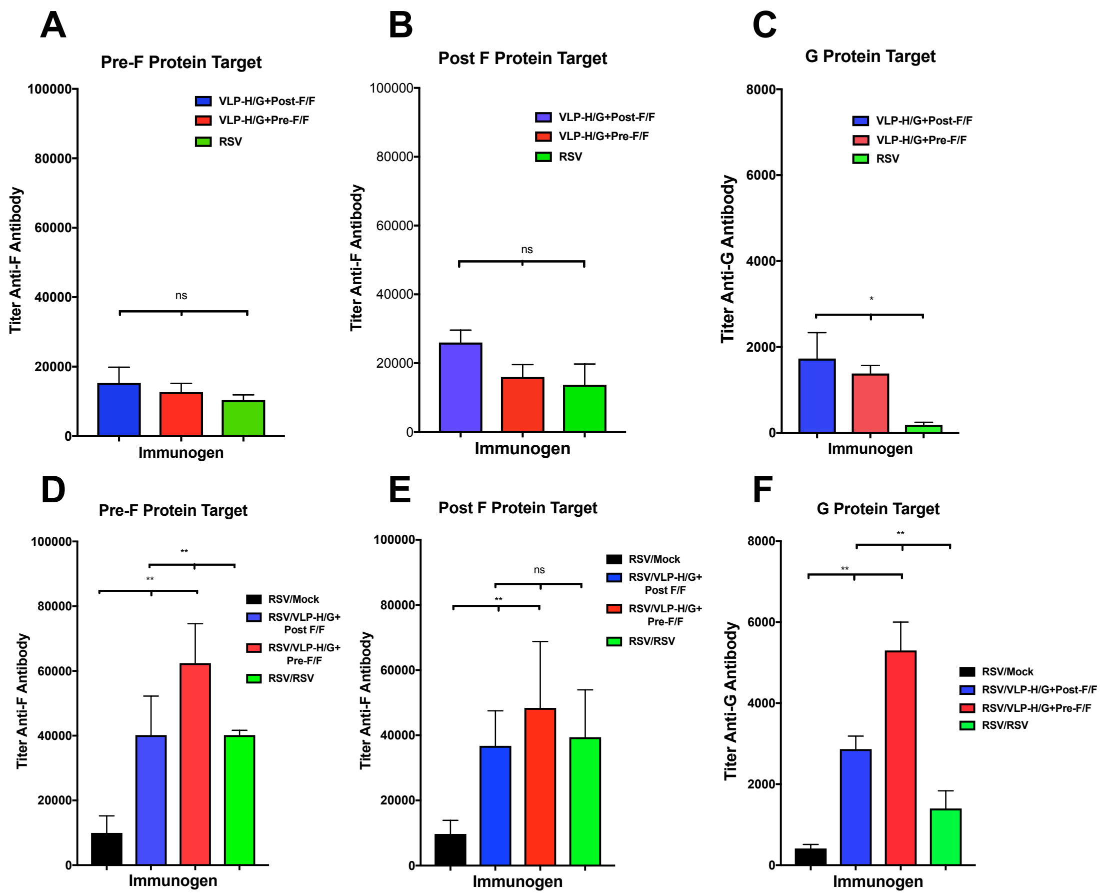

Supplementary Figure 7: *Differences in total IgG serum levels at 13 weeks*. Top panels: Concentrations of total IgG that bound to soluble (A) pre-F, (B) post-F, or (C) G protein targets at 13 weeks from the initiation of the experiment, time of maximal antibody titer, in naïve animals. Bottom panels: total IgG concentrations at 13 weeks in RSV-primed animals specific for soluble (D) pre-F, (E) post- F, or (F) G protein at the indicated times. Columns show the mean of 3 to 5 separate determinations and the *p* values comparing different pooled sera are shown at the top of each graph. Differences shown were assessed by non parametric Kruskal-Wallis test. *, p<0.05; **, p<0.005; n.s., no significant.

Supplementary Tables

Group	Fem. (n)	Prim.	Vaccination	Dose	Pups (n)	Age Ser. Coll. (d)	Age Sac. (d)	Sample collected (Analysis)
A	9	(-)	PBS	n/a	56	28	32	Lung (VT, Histo, Cyt.), Nose (VT)
B	9	(+)	PBS	n/a	31	28	32	Lung (VT, Histo, Cyt.), Nose (VT)
C	12	(-)	VLP-PreF	100 μg	76	28	32	Lung (VT, Histo, Cyt.), Nose (VT)
D	18	(+)	VLP-Pre F	100 μg	60	28	32	Lung (VT, Histo, Cyt.), Nose (VT)
E	8	(-)	VLP-Post F	100 μg	62	28	32	Lung (VT, Histo, Cyt.), Nose (VT)
F	16	(+)	VLP-Post F	100 μg	86	28	32	Lung (VT, Histo, Cyt.), Nose (VT)
G	6	(-)	Pre F	10 μg	31	28	32	Lung (VT, Histo, Cyt.), Nose (VT)
H	7	(+)	Pre F	10 μg	41	28	32	Lung (VT, Histo, Cyt.), Nose (VT)
I	6	(-)	Post F	10 μg	25	28	32	Lung (VT, Histo, Cyt.), Nose (VT)
J	5	(+)	Post F	10 μg	30	28	32	Lung (VT, Histo, Cyt.), Nose (VT)
K	5	(-)	RSV A/Long	10^5 pfu	32	28	32	Lung (VT, Histo, Cyt.), Nose (VT)
L	4	(+)	RSV A/Long	10^5 pfu	22	28	32	Lung (VT, Histo, Cyt.), Nose (VT)

Supplementary Table 1: *Table representation of the immunization groups* for cotton rat females (left, light blue) that include the number of females (n), priming status on day 0 using RSV 10^5 PFU/animal of A/Long (+/-), vaccine and vaccine dose given intramuscularly (i.m.), Pups delivered for each group are indicated (right, light green). The number of pups (n) within each group, age of serum collection and challenge (Age Ser. Coll. In days), age at sacrifice (Age Sac, in days), and samples collected for analysis are also indicated. The experiments were planed with test groups containing 8 females per groups in Experiment XV-146, 4 to 5 females in the experiment XV-176, and 5 to 7 females in the experiment XV-186. This numbers are above or equal to 5 per group as this is the historical group size for RSV infections to detect >20% differences in viral titers upon RSV challenge. For the control group (RSV i.m. immunization) in experiment XV-146, 5 females per group were used since this group was tested

in our previous publications and the results were within the expected range. Animals were separated in groups by Sigmovir Biosystems Inc.'s animal caregivers and blindly to the investigators. Animals were excluded if they did not become pregnant or if they cannibalize the litter after delivery.

Gene	Direct	Reverse
β-Actin	TACGCCAACACAGTGCTGTCT	TCTGCATCCTGTCGGCAAT
IL-6	ATGAAGTTCCTCTCCGCAAGACACT	GACCAGAGGTGATTTTCAGTAGGC
IFN-γ	CAGATGTCGGGGATCAAAAG	GTTGATGCTTTCCTGGATGG

Supplementary Table 2: Sequence of primers used for analysis of cytokine expression in the lung of pups on day 4 p.i. (Fig.4 B).
